# Supplementary figures and images for: Predicting the contribution of climate change on North Atlantic underwater sound propagation
Source: PeerJ. 2023 Oct 10;11:e16208. doi: 10.7717/peerj.16208 (PMC10573315; doi:10.7717/peerj.16208)

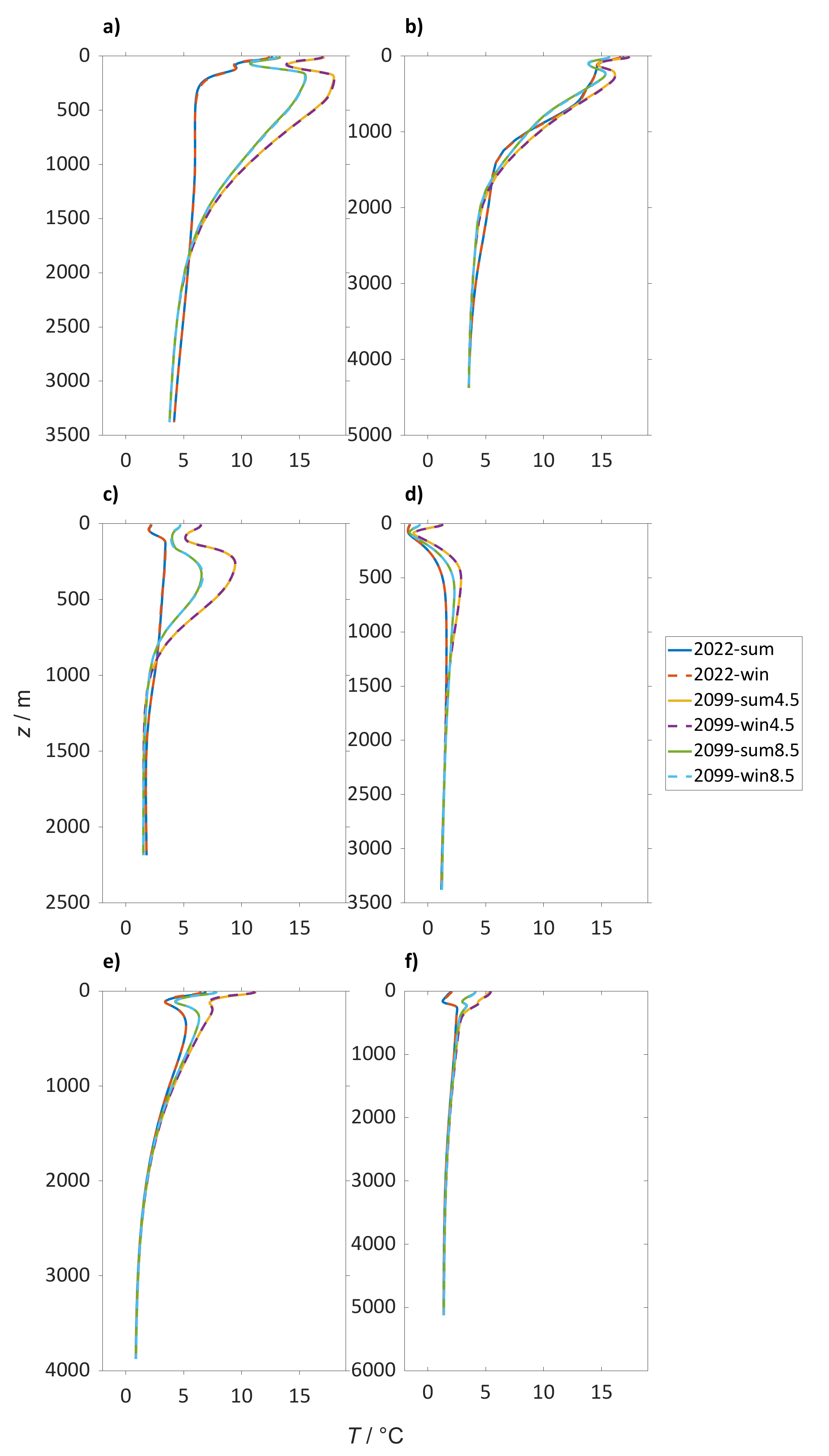

Supplement: Supplemental Information 1 — Seawater temperature (T) in ° C profiles over depth for the winter (dashed lines) and summer season (continuous lines) where in blue is boreal summer (2018 to 2022), in red winter (2018 to 2022), in yellow summer (2094 to 2098) and in purple winter (2094 to 2098) for SSP5-8.5 and green and azure for summer and winter (2094 to 2098) for SSP2-4.5 for a) Northwest Atlantic Ocean (45° N 40° W), b) Northeast Atlantic Ocean (47° N 14° W), c) Norwegian Sea (72° N 1° W), d) Arctic Ocean (75° N 140° W), e) North Pacific Ocean (50° N 167° E) and f) Southern Ocean (60° S 25° E). [file peerj-11-16208-s001.png]

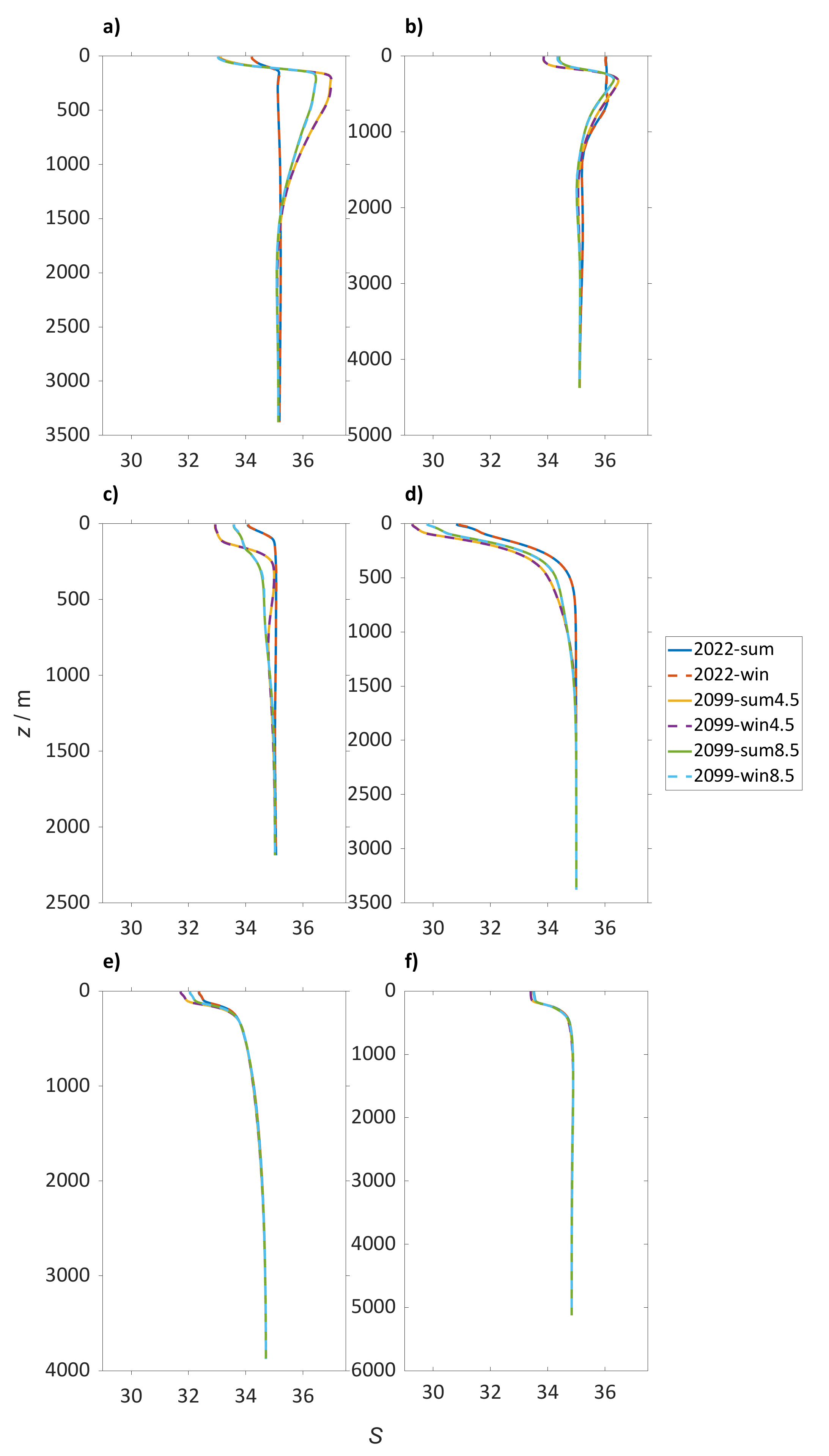

Supplement: Supplemental Information 2 — Salinity (S) profiles over depth for the winter (dashed lines) and summer season (continuous lines) where in blue is boreal summer (2018 to 2022), in red winter (2018 to 2022), in yellow summer (2094 to 2098) and in purple winter (2094 to 2098) for SSP5-8.5 and green and azure for summer and winter (2094 to 2098) for SSP2-4.5 for a) Northwest Atlantic Ocean (45° N 40° W), (b) Northeast Atlantic Ocean (47° N 14° W), (c) Norwegian Sea (72° N 1° W), (d) Arctic Ocean (75° N 140° W), (e) North Pacific Ocean (50° N 167° E) and (f) Southern Ocean (60° S 25° E). [file peerj-11-16208-s002.png]

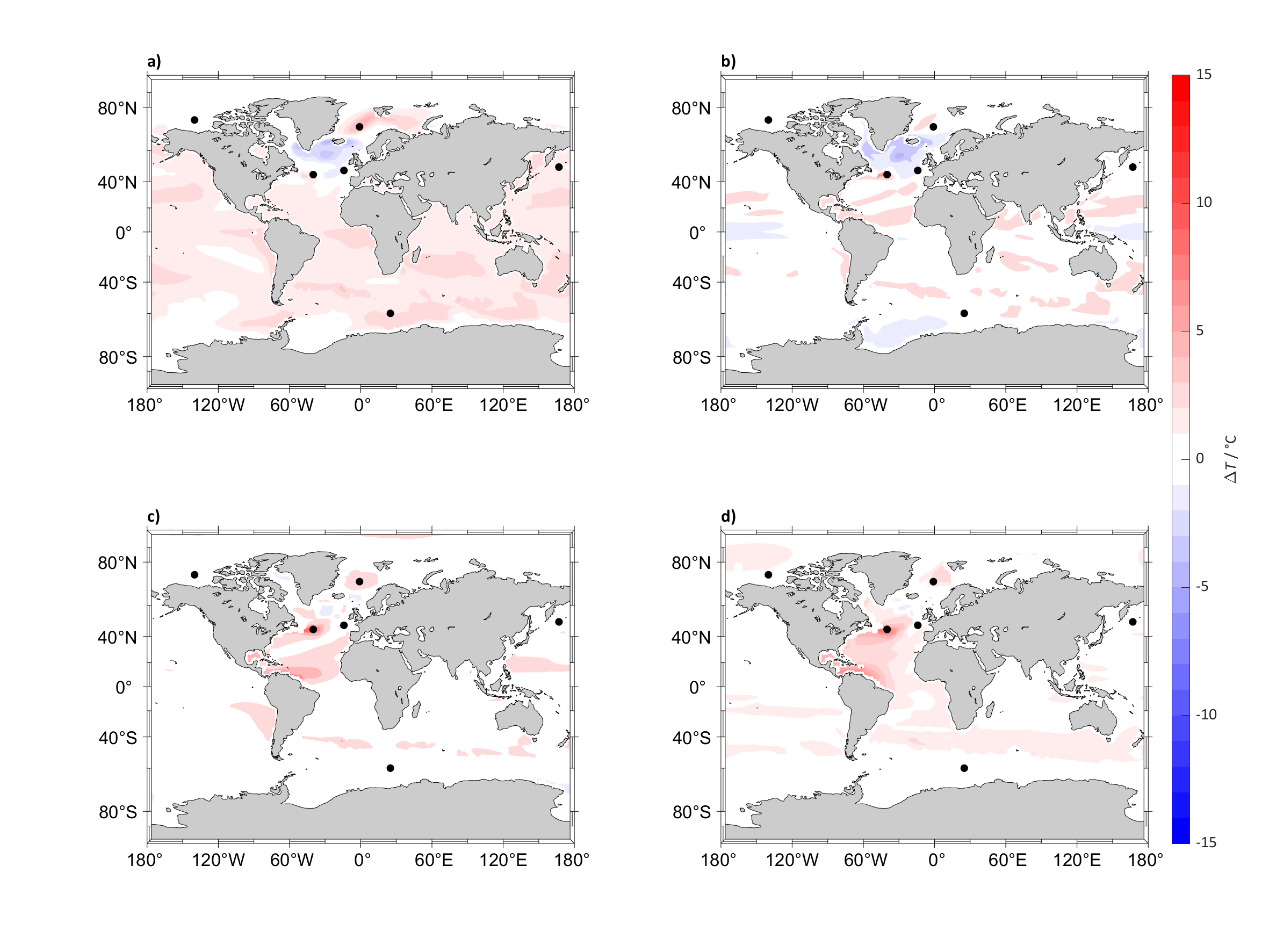

Supplement: Supplemental Information 3 — Maps of the difference in 5 years mean of seawater temperature (T) in ° C between (2018 to 2022) and (2094 to 2098) at (a) 5 m, (b) 125, (c) 300 and (d) 640 m depth calculated for SSP5-8.5. The black dots indicate the sound source locations. [file peerj-11-16208-s003.png]

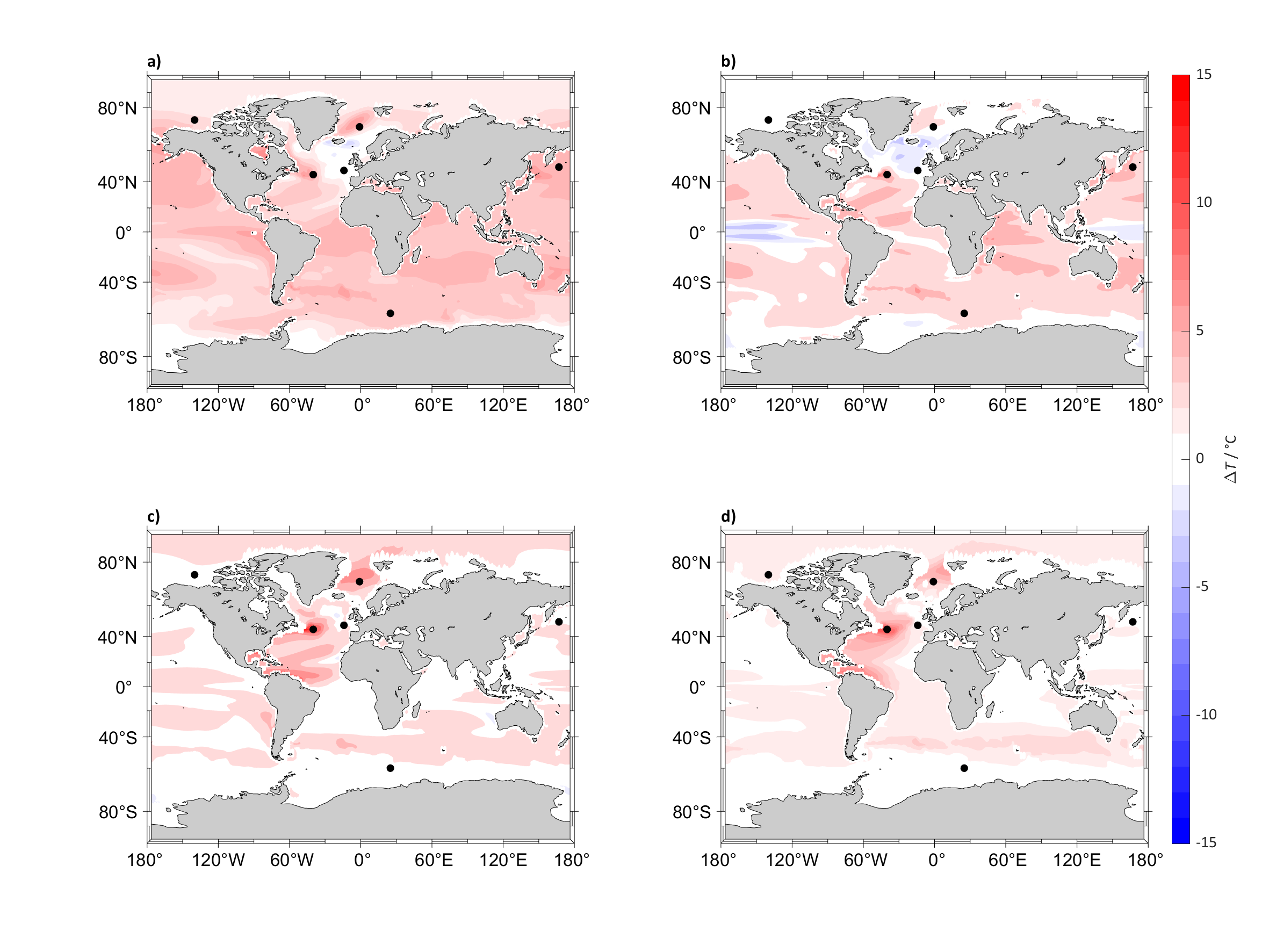

Supplement: Supplemental Information 4 — Maps of the difference in 5 years mean of seawater temperature (T) in ° C between (2018 to 2022) and (2094 to 2098) at (a) 5 m, (b) 125, (c) 300 and (d) 640 m depth calculated for SSP2-4.5. The black dots indicate the sound source locations. [file peerj-11-16208-s004.png]
